# Supplementary material for: Enhancing reliability in electrical grids: A hybrid machine learning approach for electrical faults classification
Source: PLoS One. 2026 Feb 13;21(2):e0341238. doi: 10.1371/journal.pone.0341238 (PMC12904571; doi:10.1371/journal.pone.0341238)
Supplement: Attachment — Submitted filename: Editor Response.pdf [file pone.0341238.s001.pdf]

Comment#1.

1. It is important that you include a cover letter with your manuscript. Please ensure that this letter is addressed specifically to PLoS ONE. Please also include
* why this manuscript is suitable for publication in PLoS ONE.
* how does your paper provide a worthwhile addition to the scientific literature?
* how does your paper relate to previously published work?
* which types of scientists do you believe will be most interested in your study?

Response #1

Why PLOS ONE is suitable for this research:

This work offers an interdisciplinary contribution at the intersection of electrical engineering and machine learning, aligning with PLOS ONE's focus on rigorous, impactful studies that improve practical technological systems. Our research presents findings that can directly enhance decision-making in critical infrastructure, contributing to the journal's mission of promoting high-quality scientific knowledge with real-world benefits.

How this paper adds value to the scientific literature:

Transmission line faults remain a major threat to power grid stability, and robust detection methods are essential for operational security. While many earlier works investigate individual machine learning models, comprehensive evaluations and hybrid model development are limited. Our study thoroughly compares six widely used classifiers and advances the field by proposing a novel hybrid stacking-based ensemble (RF + DT + Stacking) that significantly improves classification effectiveness. The model demonstrates strong performance, achieving 93.64% accuracy, precision, recall, and F1-score, offering a practical and more reliable solution for fault identification.

Relation to previously published work:

Existing literature primarily focuses on single-model applications or limited ensemble techniques in electrical fault detection. Our study extends these efforts by:

- Using real-world fault data rather than simulations commonly used in prior studies,
- Incorporating multiple ensemble strategies (Hard/Soft Voting, Stacking, Blending),
- Demonstrating performance improvements through a newly designed hybrid approach.

Thus, we provide both a methodological enhancement and broader experimental evaluation compared to earlier work.

Intended audience and scientific impact:

This paper will be highly relevant to scientists and professionals in the following fields:

- Electrical engineering & power systems – improving reliability and maintenance planning
- Machine learning & data-driven engineering – demonstrating practical ensemble applications
- Energy policy & infrastructure management – supporting operational resilience

Given the growing focus on smart grids and automation, we believe that this manuscript will attract a diverse audience concerned with ensuring the security and sustainability of modern energy systems.

Comment#2. Please ensure that you refer to Figure 3 in your text as, if accepted, production will need this reference to link the reader to the figure.

Response #2:

Thank you for the observation. We have now added an explicit reference to Figure 3 within the main text to ensure proper linkage during production. The manuscript has been updated accordingly.
