# Supplementary material for: Enhancing reliability in electrical grids: A hybrid machine learning approach for electrical faults classification
Source: PLoS One. 2026 Feb 13;21(2):e0341238. doi: 10.1371/journal.pone.0341238 (PMC12904571; doi:10.1371/journal.pone.0341238)
Supplement: Attachment — Submitted filename: Response-to-Reviewers.pdf [file pone.0341238.s002.pdf]

Manuscript Title: Enhancing Reliability in Electrical Grids: A Hybrid Machine Learning Approach for Electrical Faults Classification

Dear Academic Editor,
We sincerely thank you and the reviewers for your constructive and insightful comments. We have carefully revised the manuscript based on all suggestions. Below, we provide a point-by-point response to each comment and describe the corresponding changes made in the revised manuscript. All modifications are highlighted in the revised version.

Reviewer #5:

Comment #1:

The title of the paper suggests that the research is focused on "Electrical Faults Classification", but in the Introduction the authors state the following:
"This paper investigates the following key research questions:
• **RQ1:** What machine learning algorithms have been utilized for electrical fault detection, and what advantages do they offer in terms of accuracy, efficiency, and real-time fault diagnosis?
• **RQ2:** What novel methodology or framework is introduced in this study, and how does it enhance fault detection performance?
• **RQ3:** Which hybrid machine learning model can outperform previous research and improve the accuracy of electrical fault classification?"
These three questions suggest that the issues related to fault regimes in electrical networks: detection, classification, localization, and diagnosis of faults in electrical networks have the same significance.
What is the position of the authors in this regard? Does the paper address and answer all these issues?

Response:

We thank the reviewer for this insightful observation. We fully agree that the scope suggested in RQ1–RQ3 may give the impression that the paper addresses detection, classification, localization, and diagnosis equally. To avoid this confusion, we have clarified the scope and revised the research questions.

Clarification:

- This paper exclusively focuses on electrical fault classification, not detection, localization, or diagnosis.
- The dataset used is already labeled with fault types; therefore, the task is classification only.
- Fault detection (identifying whether a fault exists), fault localization (distance from the relay point), and fault diagnosis (root cause analysis) are not addressed, as they require different types of data and system measurements (e.g., PMU-based waveform signatures, traveling wave characteristics), which are beyond the dataset and scope.

Revised Research Questions:

We updated RQ1–RQ3 with the following version:

RQ1: What machine learning algorithms have been utilized for electrical fault classification, and what advantages do they offer in terms of accuracy, efficiency, and real-time fault classification?

RQ2: What novel methodology or framework is introduced in this study, and how does it enhance fault classification performance?

RQ3: Which hybrid machine learning model can outperform previous research and improve the accuracy of electrical fault classification?

Comment #2:

The authors have incompletely answered, or have avoided answering, some questions raised in previous reviews, especially related to the specifics of the application - which distinguishes it from other ML applications - otherwise the algorithm can be applied to any data set, etc.

Response:

We appreciate the reviewer's concern and have added explicit sections explaining what makes electrical fault classification domain-specific and not a generic ML application.

Clarification Provided in the Revised Manuscript:

We added a paragraph in both the Introduction and Discussion sections highlighting the domain-specific characteristics:

Newly Added Explanation (to Introduction):

Unlike general ML classification problems, electrical fault classification involves high-frequency transient behavior in voltages and currents. Fault events cause abrupt changes in current magnitudes (Ia, Ib, Ic) and subtle variations in voltage signals. These patterns are nonlinear and system-dependent, governed by power-system physics, line impedance, fault resistance, and sequence component behavior. The selected ML models are therefore applied to system-generated signals representing real electrical phenomena rather than generic tabular data.

Newly Added Explanation (to Discussion section):

Although ML techniques can theoretically be applied to any tabular dataset, electrical fault classification is unique because the features arise directly from power-system transient dynamics. The model must learn system-specific nonlinearities resulting from impedance, line length, and fault resistance, which are not present in generic classification tasks.

Closing Statement

We appreciate the time and effort invested by the editor and reviewers. Their feedback has significantly improved the quality of our manuscript. We believe the revised version now addresses all concerns thoroughly and meets the journal's standards.
