# Supplementary material for: Enhancing reliability in electrical grids: A hybrid machine learning approach for electrical faults classification
Source: PLoS One. 2026 Feb 13;21(2):e0341238. doi: 10.1371/journal.pone.0341238 (PMC12904571; doi:10.1371/journal.pone.0341238)
Supplement: Attachment — Submitted filename: Response-to-Reviewers_auresp_3.pdf [file pone.0341238.s003.pdf]

Manuscript Title: Enhancing Reliability in Electrical Grids: A Hybrid Machine Learning Approach for Electrical Faults Classification

Dear Academic Editor,

We thank the reviewer for highlighting the need to further clarify the nature of our contribution. In response, we have removed language implying algorithmic novelty and explicitly reframed the study as an application-oriented benchmarking and evaluation work, emphasizing interpretability and computational efficiency rather than methodological innovation.

We have also added a dedicated limitations paragraph acknowledging the absence of cross-validation, robustness analysis, and statistical significance testing, and we have carefully qualified claims related to generalization and real-time deployment.

Finally, we revised the discussion to avoid overemphasizing numerical accuracy and to clearly articulate the trade-off between accuracy, interpretability, and computational cost, positioning the proposed approach as a practical alternative rather than a state-of-the-art solution.

With Best Regards,

Jia Uddin, Ph.D.
Professor| *Associate Professor*
Artificial Intelligence and Big Data Department
Endicott College, Woosong University, Daejeon, Korea
Office: JCFS Building #2, 2nd Floor

Comment #1:

The claims regarding novelty have been moderated compared to the previous version, which is appreciated. Nevertheless, some statements still imply methodological novelty (e.g., references to a “novel methodology”). Please further clarify that the contribution is application-oriented, focusing on benchmarking, interpretability, and computational efficiency rather than algorithmic innovation.

Response:

We thank the reviewer for this valuable observation. We agree that some phrasing in the revised manuscript could still be interpreted as implying algorithmic novelty. The primary intention of this study is not to introduce a new learning algorithm, but rather to provide an application-oriented evaluation of classical and ensemble machine learning models for electrical fault classification, with a focus on benchmarking, interpretability, and computational efficiency.

Author’s Action:

We have removed or revised all statements implying methodological or algorithmic novelty (e.g., “novel methodology” or “novel framework”). The Abstract, Contributions section, and Research Question RQ2 have been rewritten to clearly emphasize that the contribution is application-driven, focusing on comparative analysis, practical trade-offs, and lightweight deployment rather than algorithmic innovation.

Comment #2:

The study still does not include cross-validation (e.g., k-fold CV), robustness analysis, or statistical significance testing. While execution time and memory usage have been added, these do not substitute for proper validation. Please explicitly acknowledge this limitation in the manuscript and avoid claims that may suggest strong generalization performance.

Response:

We appreciate the reviewer’s insightful comment and fully acknowledge this limitation. Due to computational and scope constraints, the current study evaluates model performance using a single train–test split. We agree that this does not provide the same level of robustness as cross-validation or statistical significance testing.

Author’s Action:

A dedicated limitations paragraph has been added to the Strengths, limitations, and Future perspectives sections explicitly stating the absence of cross-validation, robustness analysis, and statistical testing. Additionally, we have revised the manuscript to avoid language implying strong generalization or definitive robustness, and we now frame the reported results as indicative and comparative rather than conclusive.

Comment #3:

The discussion on real-time feasibility and computational efficiency has improved. However, the absence of validation on IEEE test systems or real-world data remains a limitation. Please ensure that claims regarding practical deployment are carefully qualified and framed as prospective rather than demonstrated.

Response:

We thank the reviewer for highlighting this important point. We agree that, without validation on IEEE benchmark systems or real-world operational data, claims regarding real-time deployment must be carefully qualified.

Author’s Action:

We have revised the Discussion section to explicitly state that real-time feasibility and deployment readiness are prospective rather than experimentally demonstrated. The manuscript now clarifies that additional validation on IEEE benchmark systems and real-world datasets is required before practical deployment, and this has been clearly identified as part of future work.

Comment #4:

Please avoid overemphasizing the numerical accuracy values and refrain from using language that implies state-of-the-art performance. The reported accuracy (93.64% for six-class classification) is lower than several deep learning-based methods cited in the literature. The discussion should more clearly emphasize the trade-off between accuracy, interpretability, and computational efficiency.

Response:

We appreciate the reviewer’s constructive feedback. We agree that numerical accuracy alone should not be overemphasized, particularly when deep learning-based approaches report higher performance under different assumptions and resource requirements.

Author’s Action:

We have revised the Discussion and Conclusion sections to remove any language implying state-of-the-art performance. The manuscript now explicitly acknowledges that deep learning methods achieve higher accuracy, while emphasizing the trade-off achieved by the proposed approach in terms of interpretability, reduced computational cost, and suitability for resource-constrained environments. Accuracy is now presented as competitive rather than superior.
